# Effectiveness of Early Mobilization and Bed Positioning in the Management of Muscle Weakness in Critically Ill People Under Invasive Mechanical Ventilation in Intensive Care: A Systematic Review of Intervention Literature Protocol

**DOI:** 10.3390/nursrep15030075

**Published:** 2025-02-20

**Authors:** Inês Bento, Bruno Ferreira, Cristina Lavareda Baixinho, Maria Adriana Henriques

**Affiliations:** 1 Nursing Department, Red Cross Higher School of Health, 1300-125 Lisbon, Portugal; 2 Nursing School of Lisbon, University of Lisbon, 1649-004 Lisbon, Portugal; 3 Nursing Research, Innovation and Development Centre of Lisbon (CIDNUR), 1900-160 Lisbon, Portugal; 4 School of Health, Polytechnic Institute of Setubal, 2910-761 Setúbal, Portugal; 5 Rehabilitation Nursing Department, Nursing School of Lisbon, 1600-190 Lisbon, Portugal; 6 Community Health Department, Nursing School of Lisbon, 1600-190 Lisbon, Portugal

**Keywords:** early mobilization, rehabilitation, muscle weakness, critical care nursing, activities of daily living, functional status

## Abstract

**Background**: Post Intensive Care Syndrome (PICS) is a set of physical, cognitive, and mental health symptoms that arise following intensive care (ICU) hospitalization. Regarding physical changes, muscle weakness is highlighted, potentially leading to functional impairments during and after hospitalization. Multidisciplinary guidelines recommend early mobilization, a rehabilitation intervention, as a strategy to prevent ICU-acquired muscle weakness and reduce functionality impairments. **Objective**: This study aims to evaluate the effectiveness of early mobilization and positioning interventions to prevent or minimize ICU-acquired weakness in critically ill patients under invasive mechanical ventilation (IMV). **Methods**: A systematic review of effectiveness will be conducted following Cochrane recommendations. Searches will be made in MEDLINE (via PubMed), CINAHL, Scopus, and Web of Science. Eligible studies will include randomized controlled trials on the functional management of muscle weakness, muscle strength, and ICU-acquired muscle weakness in adults (≥18 years) who have undergone IMV. Eligible interventions (and comparators) include any manual mobilization and positioning strategy or the use of medical devices. Two reviewers will independently select studies, extract data using a piloted tool and assess bias with the RoB 2 tool. If appropriate, a meta-analysis will be conducted, pooling standardized mean differences using a random-effects model. **Results**: This review included primary experimental studies manipulating at least one variable, control group studies, or randomized trials comparing early intervention protocols, programs, or plans with standard care or existing approaches in the ICU. **Conclusions**: This review will provide meaningful comparisons of different mobilization and positioning strategies, evaluating their impact on muscle strength and functionality in critically ill patients. Systematic Review Registration: PROSPERO CRD4202348091.

## 1. Introduction

Hospitalization in an intensive care unit (ICU) can lead to the development of a set of signs and symptoms known as Post Intensive Care Syndrome (PICS). This syndrome can occur in three different dimensions of the person in critical condition (PCC), namely physical, cognitive and mental health. PCC family can also have mental health symptoms, like anxiety and depression feelings: Post Intensive Care Syndrome—Family (PICS—F) [1,2].

In the field of physical problems, intensive care unit-acquired weakness (ICUAW) is prevalent in more than 70% of some PCC sub-groups [3] and is an increasingly common complication of critical illness, with an annual incidence of 25 to 31% [4]. It underpins the impaired functional capacity observable during and after a stay in intensive care. In the short term, it increases hospital mortality, and, in the long term, it decreases quality of life and increases the use of health resources and their costs, being associated with a negative impact on the lives of the family and/or caregivers [4]. ICUAW is clinically appropriate when there is no other reason for the onset of muscle weakness other than intensive care admission.

Clinically, critical illness compromises the mobility of the PCC, contributing to the appearance of muscle weakness, but physiologically it is not based solely on muscle disuse. It is characterized by acute loss of skeletal muscle and consequent impairment of its contractile function [5].

There are many risk factors involved in inducing muscle dysfunction, such as compromised mobility, systemic and intramuscular inflammation, oxidative stress and neurological damage [3].

The drugs used to control pain and manage sedation, and which are also needed for muscle blockade, cause a decrease in muscle electrical conduction, strongly contributing to the appearance of muscle weakness [3].

Muscle weakness prolongs the duration of invasive mechanical ventilation (IMV), but studies show that people who undergo more than 48 h of IMV show a rapid loss of skeletal muscle, which affects their clinical course during hospitalization [5,6].

It is estimated that muscle loss occurs between 25 and 50% in PCCs under IMV during 14 days of hospitalization, which corresponds to a loss of muscle mass of around 4% per each day of IMV [3].

As a result of this neuromuscular damage, there are functional changes and, consequently, a loss of ability to carry out the activities of daily living. This can alter the ability to live independently, leading to the need for help from others to carry out activities such as walking, bathing or transferring [7]. At the same time, loss of muscle strength, decrease in joint range and dependence on walking contribute to the appearance of numerous complications associated with immobility syndrome [8,9], such as an increased prevalence of infections, pressure ulcers, falls [10] and social isolation [9].

Around 50% of intensive care survivors who have been under prolonged IMV need assistance from a caregiver until around 1 year after discharge [11]. There is evidence of an increase in the use of health resources and their costs, as well as a negative impact on the life of the PCC after discharge and that of their family [11].

Current guidelines for clinical practice in intensive care, which include the prevention and treatment of immobility [12], recognize physical rehabilitation as a preventive and therapeutic measure for physical impairments caused by PICS. Mobilization is a type of rehabilitation intervention that facilitates joint movement and promotes energy expenditure, showing positive results in enhancing functionality and the psychological well-being of critically ill patients. In intensive care, mobilization has been studied in the early phase of the disease process. Initial mobilization interventions include passive mobilization of all four limbs, progressing to active-assisted exercises as the patient improves and cooperates. Throughout the clinical trajectory, mobilization exercises can be performed either in bed or out of bed and may involve medical devices [13]—equipment that promotes joint movement and muscular activation. Among these, the cycle ergometer can be identified (a device that allows the patient to perform repetitive low-resistance movements), as well as neuromuscular electrical stimulation (NMES) (which employs equipment to assist in generating functionally useful muscle contractions, activating not only local musculature but also reflex mechanisms necessary for the reorganization of motor activity) [13]. Several studies combine different mobilization strategies, leading to the development of mobilization programs, plans or protocols aimed at preventing or minimizing ICUAW [14,15].

Early mobilization aims to reduce the time spent immobilized in bed, providing joint movement to the critically ill patient as early as possible [12,13]. However, the literature lacks consensus regarding the term “early” in this context. Some studies define early mobilization as beginning within 48 to 72 h after ICU admission. Nevertheless, patients under invasive mechanical ventilation (IMV) have shown ICU-acquired muscle weakness as early as at 48 h of ventilation [13]. The initiation of mobilization as a rehabilitation intervention within the first 24 h has been found to pose viability issues, as patients often do not demonstrate sufficient clinical stability for the intervention to be conducted safely [13]. For the purpose of this review, mobilization will be considered early if initiated between 24 and 72 h after ICU admission.

Given the multiple approaches described in the literature regarding early mobilization interventions for the prevention of ICUAW, it is essential to determine which strategies are the most effective. More specific recommendations are needed in this field, as studies assess different types of interventions at varying time points to determine the optimal initiation of rehabilitation in an ICU [12,13].

Considering the above, the objective of this study is to evaluate the effectiveness of early mobilization and positioning interventions to prevent or minimize ICUAW in critically ill patients under IMV.

## 2. Materials and Methods

The present protocol is for an effectiveness systematic review, which will be carried out according to Cochrane Guidelines for Systematic Review of Interventions [16] and designed according to the recommendations of the Preferred Reporting Items for Systematic Review and Meta-Analysis (PRISMA) Protocols checklist, PRISMA-P (Preferred Reporting Items for Systematic review and Meta-Analysis Protocols) [17]. According to Cochrane, the protocol should be drawn up in accordance with the following steps: planning the search, planning the assessment of risk of bias in the included studies, planning the synthesis of the results, planning the sub-group of analyses and planning the GRADE assessment and “summary of findings” table [18].

### 2.1. Research Question

To answer the research question in the PICOs format, what is the effectiveness of early mobilization and bed positioning interventions for critically ill people in the ICU in regard to reducing muscle weakness associated with intensive care?

P (Population): Adult people who are, or have been in a critical situation under IMV.

I (Intervention) protocols: Programs or clinical intervention plans that integrate any manual mobilization and positioning strategy, or medical device, started early (24–72 h).

C (Comparator) protocols: Programs or clinical intervention plans that integrate any assumption of the aforementioned intervention, and the programs may be comparable to each other; isolated mobilization and/or positioning interventions delivered without integrating any program, plan or protocol.

O (outcomes): The presence of acquired muscle weakness in the ICU; muscle strength and functional status at discharge from the ICU.

s (study design): Randomized controlled trials will be included.

### 2.2. Eligibility Criteria

Each element of the PICOs acronym guided the definition of each specific inclusion criteria, presented in Table 1.

### 2.3. Study Design

Any randomized control trial (RCT) reporting adult patients aged 18 or older who are or have been under IMV, muscle strength or functional status after implementing an early mobilization program/protocol/plan or bed positioning that employs manual mobilization or the use medical device, such as a cycle ergometer, neuromuscular electrical stimulation (NMES) or a verticalization plan. The intervention can be performed in bed or out of bed.

#### 2.3.1. Population

Studies that include adult patients aged 18 or older who are or have been under IMV.

#### 2.3.2. Interventions

The strategies to reduce or prevent muscle weakness associated with intensive care hospitalization are related to programs, protocols, or intervention plan implementations that include any manual mobilization and positioning strategy or which use medical devices. They can include the mobilization of joints, progressive verticalization and simulation of orthostatism and/or neuromuscular stimulation in 24–72 h after ICU admission.

#### 2.3.3. Comparator

The comparative intervention should incorporate one of the following assumptions:

- Involvement of a program, protocol, or intervention plan that includes any of the aforementioned interventions;
- Integration of mobilization care for PCCs in intensive care, in bed or out of bed, using manual therapy and available medical devices, without any established protocol, program or plan at any stage of the critical illness;
- Mobilization or exercise that begins only when the PCC is transferred from the ICU;
- Exercise or training that only considers the respiratory muscles or is aimed solely at respiratory-functional re-education;
- Inclusion of positioning in bed, or alternating decubitus, without any established protocol, program or plan and at any stage of the critical illness.

#### 2.3.4. Outcomes

Functional status, irrespective of the tool used; muscle strength (Medical Research Council score); presence of muscle weakness acquired in intensive care (Medical Research Council sum-score); length of stay (ICU and hospital stay); number of days under IMV; critical illness severity classification (APACHE) score; sequential organ failure assessment (SOFA) score; mortality.

### 2.4. Research Strategy

A search will be carried out in the MEDLINE (via PUBMED), CINAHL, Scopus and Web of Science databases.

The search strategy was developed using both Medical Subject Headings (MeSH) and free-text terms likely to appear in the title, abstract or full text of the literature. Search terms were carefully selected to make sure the net was wide enough to include all the relevant publications. The main search terms were rehabilitation, early mobilization, and intensive care. For the intervention, in order to identify programs, protocols, or plans, terms such as manual mobilization and positioning strategy or use of medical devices started early were used. Natural language terms and descriptors were employed to ensure the inclusion of studies related to positioning, in bed mobilization, early mobilization out of bed, and physical activity. The search was limited between the years 2012–2024.

A total of 49 articles were identified from MEDLINE (Table 2), 2 articles from CINAHL, 391 articles from Scopus and 77 articles from Web of Science.

### 2.5. Selection Process

Two review authors will independently assess all the studies identified as a result of the search strategy. In the event of disagreement, the third review author will be consulted, if necessary.

### 2.6. Assessment of Methodological Quality and Risk of Bias

The assessment of the risk of bias of the included studies will take into account the potential sources of bias and may determine a low risk of bias (if all criteria were fulfilled), moderate risk of bias (one or more criteria were partially fulfilled), high risk of bias (one or more criteria were not fulfilled) and unclear (not enough information is available to assess the study with regard to the risk of bias). The Cochrane Risk of Bias Assessment Tool (Rob 2) [16] will be applied.

### 2.7. Data Synthesis

The synthesis of the information regarding the characteristics and results of the included studies will be presented in a table and complemented with a narrative summary, which will assess the methods used, and the results of the studies.

If the data allows, a meta-analysis will be carried out. If the sample is heterogeneous, a narrative synthesis will be drawn up.

The principles of the GRADE system [18] will be used to assess the quality of the body of evidence associated with the conclusions presented. A table of results will be constructed according to a level of evidence approach.

The results of the systematic review will be reported according to the PRISMA-P guidelines [17].

### 2.8. Ethical Issues

The systematic literature review is a secondary study, but it will follow all the principles of scientific integrity and rigorous writing.

## 3. Discussion

Studies have shown that initiating early mobilization after a patient’s admission to the ICU significantly reduces the incidence of ICUAW, delirium, length of ICU and hospital stay, as well as days on mechanical ventilation. Despite these well-documented benefits, an effective and standardized method for early mobilization remains to be developed [19,20]. The primary outcome of this systematic review is to contribute to the development of intervention programs that demonstrate efficacy in the prevention and mitigation of ICUAW.

ICUAW is an early-onset and clinically significant issue for critically ill patients. Its pathophysiology is complex, involving multiple organ systems, but it is fundamentally characterized by muscle dysfunction [3]. This dysfunction results from structural and physiological changes in muscle tissue, alterations in cellular electrical excitability and mitochondrial dysfunction [5]. Clinically, ICUAW manifests as varying degrees of limb muscle weakness while preserving the function of muscles responsible for facial expression [21].

The PADIS Guidelines for clinical practice highlight the importance of early mobilization in rehabilitation programs for critically ill patients. These guidelines emphasize that early mobilization should be systematically integrated into ICU rehabilitation care rather than being replaced by standard care or delayed interventions with shorter duration and reduced frequency [17]. However, no consensus exists regarding the type and duration of exercises required to optimize recovery.

Recent studies confirm that early mobilization has a significant impact on reducing muscle weakness at ICU discharge [15,17]. The benefits extend beyond the ICU stay, enhancing long-term functionality [5] and reducing the overall duration of mechanical ventilation [15,17]. Moreover, early mobilization is associated with shorter hospital stays and improved recovery trajectories [5].

Despite these advantages, critically ill patients remain vulnerable to long-term impairments due to PICS, which includes physical, cognitive, and psychological dysfunctions. PICS affects a substantial proportion of ICU survivors, impacting their ability to regain independence and return to daily activities [22]. The study highlights that muscle weakness, fatigue and cognitive decline often co-occur, further complicating rehabilitation efforts. Additionally, research from the ARDSNet Long-Term Outcomes Study (ALTOS) suggests that prolonged inflammation during critical illness may contribute to persistent functional limitations [22].

Given the diverse approaches to early mobilization interventions in the literature, it is essential to determine which strategies are most effective in preventing ICUAW. Current evidence underscores the need for specific clinical recommendations, as different studies assess various mobilization protocols at different time points to establish the optimal timing and modality of ICU rehabilitation [12]. This review will strengthen the evidence base, providing insights into early mobilization interventions and their effects on muscle strength, functional status in daily activities and the prevalence of ICUAW.

By identifying and analyzing the most effective mobilization strategies, this study can contribute to the development of standardized rehabilitation protocols, ultimately optimizing the quality of care provided to critically ill patients.

## 4. Conclusions

The results of this review will enable meaningful comparisons between different mobilization and positioning strategies in regard to their impact on muscle strength and functionality in critically ill patients.

The evidence of the effectiveness of these interventions will facilitate clinical guidance and support the training of healthcare professionals in this field.

## Figures and Tables

**Table 1:** Eligibility criteria according to the PICOs acronym. Lisbon, 2024.

Selection Criteria	Inclusion Criteria	Exclusion Criteria
Population (P)	Adults aged ≥ 18 years, admitted to intensive care who are, or have been, under invasive mechanical ventilation.	Adults aged ≥ 18 years, admitted to intensive care with no invasive ventilation required.
Intervention (I)	- Programs, protocols or intervention plan implementations that include any manual mobilization and positioning strategy or which use medical devices started early (24–72 h).	- Mobilization or bed positioning intervention without any established protocol, program or plan, at any stage of the critical illness; - Exercise or training that only considers the respiratory muscles or is aimed solely at respiratory-functional re-education; - Mobilization or exercise that begins only when the PCC is transferred from the ICU. - Mobilization or exercise that begins in UCI after 72 h of the UCI admission. - Interventions aimed to people in a critical condition who have not received invasive mechanical ventilation.
Comparator (C)	- Programs, protocols or intervention plan implementations that include any manual mobilization and positioning strategy or which use medical devices started early. - Mobilization or bed positioning intervention without any established protocol, program or plan, at any stage of the critical illness. - Exercise or training that only considers the respiratory muscles or is aimed solely at respiratory-functional re-education. - Mobilization or exercise that begins only when the PCC is transferred from the ICU.	- Interventions aimed at people in a critical condition who have not received invasive mechanical ventilation.
Outcomes (O)	- Functional status, muscle strength, presence of muscle weakness acquired in intensive care, ICU and hospital length of stay, number of days under IMV, critical illness severity classification (APACHE) score; sequential organ failure assessment (SOFA) score; mortality.	- All the results related to other dimentions, such as: cognitive, psychological and mental-health.
**Study Design (s)**	Randomized controlled studies.	Studies which are not randomized.

**Table 2:** Search Strategy on MEDLINE (via PUBMED); Lisbon, 2024.

	Search Strategy	Number of Articles
# 1	(“person*”[Title/Abstract] OR “adult”[MeSH Terms] OR “young adult”[MeSH Terms] OR “old adult*”[Title/Abstract] OR “patient*”[Title/Abstract] OR “patients”[MeSH Terms]) AND 2012/01/01:2025/12/31[Date-Publication]	6,075,835
# 2	(“intensive care units”[MeSH Terms] OR “critical care”[MeSH Terms] OR “critical illness”[MeSH Terms] OR “ICU”[Title/Abstract] OR “intensive care unit*”[Title/Abstract] OR “critical care”[Title/Abstract] OR “critical illness”[Title/Abstract]) AND (2012:2024[pdat])	189,435
# 3	(“patient positioning”[MeSH Terms] OR “supine position”[MeSH Terms] OR “dorsal position”[Title/Abstract] OR “supin*”[Title/Abstract] OR “prone position”[MeSH Terms] OR “prone”[Title/Abstract] OR “sitting position”[MeSH Terms] OR “sitting”[Title/Abstract] OR “lateral*”[Title/Abstract] OR “activities of daily living”[MeSH Terms] OR “early mobilization”[Title/Abstract] OR “continued mobilization”[Title/Abstract] OR “early mobility bundle”[Title/Abstract] OR (“patient*”[All Fields] AND “rising”[Title/Abstract]) OR “early rehabilitation”[Title/Abstract]) AND (2012:2025[pdat])	385,173
# 4	(“Exercise”[MeSH Terms] OR “rehabilitation”[MeSH Terms] OR “early mobilization”[Title/Abstract] OR “exercise therapy”[Title/Abstract] OR “early ambulation”[MeSH Terms] OR “rehabilitation nursing”[MeSH Terms] OR “physical therapy modalities”[MeSH Terms] OR “endurance training”[Title/Abstract] OR “resistance training”[Title/Abstract] OR “physical stimulation”[MeSH Terms] OR “electric stimulation therapy”[MeSH Terms] OR “rehabilitation program”[Title/Abstract]) AND (2012:2024[pdat])	386,364
# 5	(“paresis”[MeSH Terms] OR “muscle weakness”[MeSH Terms] OR “muscle strength”[MeSH Terms] OR “muscle strength dynamometer”[MeSH Terms] OR “functional status”[MeSH Terms] OR “functional independence”[Title/Abstract] OR “functional dependence”[Title/Abstract]) AND (2012:2024[pdat])	49,618
# 6	(“randomized controlled trials as topic”[MeSH Terms] OR (“controlled”[All Fields] AND “clinical trials as topic”[MeSH Terms]) OR “randomized”[Title/Abstract] OR “randomized controlled trial”[Publication Type]) AND 2012/01/01:2025/12/31[Date-Publication]	603,394
#1 AND #2 AND #3 AND #4 AND #5 AND #6	(“person*”[Title/Abstract] OR “adult”[MeSH Terms] OR “young adult”[MeSH Terms] OR “old adult*”[Title/Abstract] OR “patient*”[Title/Abstract] OR “patients”[MeSH Terms]) AND 2012/01/01:2025/12/31[Date-Publication] AND ((“intensive care units”[MeSH Terms] OR “critical care”[MeSH Terms] OR “critical illness”[MeSH Terms] OR “ICU”[Title/Abstract] OR “intensive care unit*”[Title/Abstract] OR “critical care”[Title/Abstract] OR “critical illness”[Title/Abstract]) AND 2012/01/01:2024/12/31[Date-Publication]) AND ((“patient positioning”[MeSH Terms] OR “supine position”[MeSH Terms] OR “dorsal position”[Title/Abstract] OR “supin*”[Title/Abstract] OR “prone position”[MeSH Terms] OR “prone”[Title/Abstract] OR “sitting position”[MeSH Terms] OR “sitting”[Title/Abstract] OR “lateral*”[Title/Abstract] OR “activities of daily living”[MeSH Terms] OR “early mobilization”[Title/Abstract] OR “continued mobilization”[Title/Abstract] OR “early mobility bundle”[Title/Abstract] OR (“patient*”[All Fields] AND “rising”[Title/Abstract]) OR “early rehabilitation”[Title/Abstract]) AND 2012/01/01:2025/12/31[Date-Publication]) AND ((“Exercise”[MeSH Terms] OR “rehabilitation”[MeSH Terms] OR “early mobilization”[Title/Abstract] OR “exercise therapy”[Title/Abstract] OR “early ambulation”[MeSH Terms] OR “rehabilitation nursing”[MeSH Terms] OR “physical therapy modalities”[MeSH Terms] OR “endurance training”[Title/Abstract] OR “resistance training”[Title/Abstract] OR “physical stimulation”[MeSH Terms] OR “electric stimulation therapy”[MeSH Terms] OR “rehabilitation program”[Title/Abstract]) AND 2012/01/01:2024/12/31[Date-Publication]) AND ((“paresis”[MeSH Terms] OR “muscle weakness”[MeSH Terms] OR “muscle strength”[MeSH Terms] OR “muscle strength dynamometer”[MeSH Terms] OR “functional status”[MeSH Terms] OR “functional independence”[Title/Abstract] OR “functional dependence”[Title/Abstract]) AND 2012/01/01:2024/12/31[Date-Publication]) AND ((“randomized controlled trials as topic”[MeSH Terms] OR (“controlled”[All Fields] AND “clinical trials as topic”[MeSH Terms]) OR “randomized”[Title/Abstract] OR “randomized controlled trial”[Publication Type]) AND 2012/01/01:2025/12/31[Date-Publication])	49

* Wildcard used for **truncation**, allowing the retrieval of multiple word variations with the same root.

## Data Availability

Data are available only upon request to the authors.

## Public Involvement Statement

There was no public involvement in any aspect of this research.

## Guidelines and Standards Statement

This manuscript was drafted against the Preferred Reporting Items for Systematic Review and Meta-Analysis (PRISMA-P) 2015 statement for systematic reviews and the meta-analysis protocol.

## Abbreviations

The following abbreviations are used in this manuscript:

CINAHL	Cumulative Index to Nursing and Allied Health Literature
GRADE	Grading of Recommendation, Assessment, Development and Evaluation
ICU	Intensive care unit
ICUAW	Intensive care unit-acquired weakness
IMV	Invasive mechanical ventilation
NMES	Neuromuscular electrical stimulation
PCC	Person in critical condition
PICS	Post Intensive Care Syndrome
PICS-F	Post Intensive Care Syndrome Family
PROSPERO	International Prospective Register of Systematic Reviews
RCT	Randomized Control Trial
RoB 2	Risk of Bias 2.0
